# Ultrawideband high density polymer-based spherical array for real-time functional optoacoustic micro-angiography

**DOI:** 10.1038/s41377-025-01894-y

**Published:** 2025-07-07

**Authors:** Pavel V. Subochev, Xosé Luís Deán-Ben, Zhenyue Chen, Maxim B. Prudnikov, Vladimir A. Vorobev, Alexey A. Kurnikov, Anna G. Orlova, Anna S. Postnikova, Alexey V. Kharitonov, Mikhail D. Proyavin, Roman I. Ovsyannikov, Anatoly G. Sanin, Mikhail Y. Kirillin, Francisco Montero de Espinosa, Ilya V. Turchin, Daniel Razansky

**Affiliations:** 1 Institute of Applied Physics, Russian Academy of Sciences, Nizhny Novgorod, Russia; 2 Institute of Pharmacology and Toxicology and Institute for Biomedical Engineering, Faculty of Medicine, University of Zurich, Zurich, Switzerland; 3 Institute for Biomedical Engineering, Department of Information Technology and Electrical Engineering, ETH Zurich, Zurich, Switzerland; 4 Instituto de Tecnologías Físicas y de la Información—CSIC, Madrid, Spain; 5 Institute for Biological and Medical Imaging, Helmholtz Center Munich, Neuherberg, Germany; 6 Present Address: Institute of Precision Optical Engineering, School of Physics Science and Engineering, Tongji University, Shanghai, China

**Keywords:** Imaging and sensing, Photonic devices

## Abstract

Owing to its unique ability to capture volumetric tomographic information with a single light flash, optoacoustic (OA) tomography has recently demonstrated ultrafast imaging speeds ultimately limited by the ultrasound time-of-flight. The method’s scalability and the achievable spatial resolution are yet limited by the narrow bandwidth of piezo-composite arrays currently employed for OA signal detection. Here we report on the first implementation of high-density spherical array technology based on flexible polyvinylidene difluoride films featuring ultrawideband (0.3–40 MHz) sub mm^2^ area elements, thus enabling real-time multi-scale volumetric imaging with 22–35 µm spatial resolution, superior image fidelity and over an order of magnitude signal-to-noise enhancement compared to piezo-composite equivalents. We further demonstrate five-dimensional (spectroscopic, time-resolved, volumetric) imaging capabilities by visualizing fast stimulus-evoked cerebral oxygenation changes in mice and performing real-time functional angiography of deep human micro-vasculature. The new technology thus leverages the true potential of OA for quantitative high-resolution visualization of rapid bio-dynamics across scales.

## Abstract (web summary)

This work reports on the first implementation of ultrawideband high density spherical detection array based on flexible polyvinylidene difluoride films for real time functional 3D optoacoustic micro-angiography

## Graphical abstract

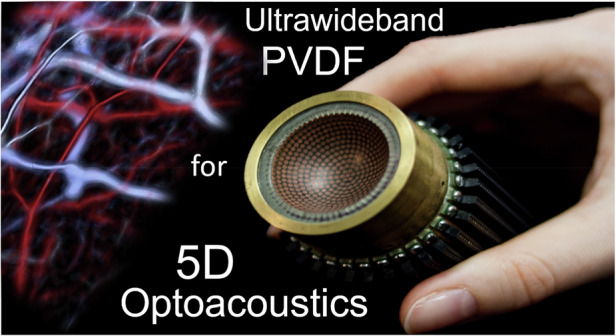

## Introduction

Optoacoustic (OA) imaging has successfully been employed to visualize biological processes across micro-, meso-, and macro-scopic domains in living organisms with temporal imaging scales ranging from sub-milliseconds to hours^1–3^. The unique multi-scale imaging capacity of the method is facilitated by the simultaneous excitation of ultrawideband ultrasound waves via instantaneous absorption of nanosecond-duration pulses of light across the imaged tissue volume. The ability to accurately map the generated ultrasound wavefield largely determines the achievable spatio-temporal resolution performance of the OA imaging system and its ability to accurately reconstruct structures of different sizes and orientations^4^. By employing tomographic detection arrays to simultaneously collect the generated pressure signals at multiple locations, it was shown possible to render OA images with a single laser pulse (single-shot excitation)^5^, thus achieving ultrafast volumetric frame rates up to the kHz range^6^, unprecedented among other bio-imaging modalities.

At present, the commonly employed piezo-composite (-ceramic) arrays are limited by their relatively narrow detection bandwidth, making them unsuitable for resolving structures across different scales^7,8^. It also turns technically challenging to scale down the element size, especially when implementing high-density piezo-composite arrays on curved geometries optimally suited for volumetric OA imaging^9,10^, which leads to poor sensitivity and lack of scalability for high-resolution imaging. In fact, sensitivity represents another critical parameter for OA imaging systems that often cope with very weak signal levels^8^. Other technologies, such as those based on capacitive micro-machined ultrasound transducers (CMUTs)^11,12^, suffer from similar limitations when attempting to realize high-frequency arrays on curved surfaces. Recently, different methods for optical ultrasound detection have shown promise for miniaturization further achieving wide bandwidth extending up to 40 MHz^13,14^. However, their commonly employed line-by-line scanning approach results in prolonged image acquisitions, hindering their application for high-speed volumetric OA imaging. Despite significant progress in parallelizing interferometric ultrasound detection systems^15^, image acquisition times remain on order of seconds thus complicating real-time functional (spectroscopic) measurements and imaging of fast biological dynamics. Besides, optical sensors commonly require applying pressure against the skin, which may result in partial collapse of small superficial vessels and altered microvascular appearance^16^.

Polyvinylidene difluoride (PVDF) films represent a flexible non-resonant alternative for the fabrication of ultrasound transducers. PVDF films exhibit a higher induced electric field per unit stress (g33 piezoelectric constant) as compared to piezo-ceramics^17^. Other key advantages, such as wideband response and acoustic impedance close to biological tissues (~2.7 MRayl), make PVDF particularly suitable for OA signal detection^18–20^. Detection bandwidths beyond 100 MHz have been demonstrated with this technology^21,22^. As the low cut-off frequency is chiefly determined by input impedance of the readout amplification circuit, sensitive detection can potentially be extended into the sub-MHz band to facilitate detection of low frequency OA signal content, which is paramount for rendering accurate and quantifiable reconstructions^23,24^. Due to the relatively low capacitance (~pF/mm^2^) of the films, large area PVDF detectors can readily be devised in a variety of configurations, from single-element spherically focused transducers for scanning OA microscopy applications^25,26^ to linear^27,28^, ring^29^ and planar^30^ arrays. The superior detection bandwidth of PVDF sensors results in an enhanced sensitivity for certain frequencies, which in turn facilitates visualizing vascular structures of the corresponding dimensions^31^. However, fabrication of high-density miniature arrays has so far been impeded by the high electrical impedance (»1 kΩ) and low capacitance of small-sized PVDF elements, necessitating a high level of miniaturization and close integration of multi-channel pre-amplification interfaces to avoid signal attenuation and bandwidth contraction due to capacitive loading.

Here we report on the first development of a spherical PVDF matrix array of 512 densely distributed sub-mm^2^ area elements tightly integrated with high channel count amplification electronics to provide a sensitive tomographic detection of OA signals over ultrawide bandwidth approaching 40 MHz. The broad detection bandwidth and angular coverage of the spherical transducer array further enable capturing more quantitative volumetric tomographic information from the imaged object across multiple spatial dimensions as compared to the piezo-composite-based array equivalents, thus efficiently exploiting the multi-scale nature of the OA method.

## Results

### The ultrawideband polymer-based spherical matrix array

**Fig. 1:**
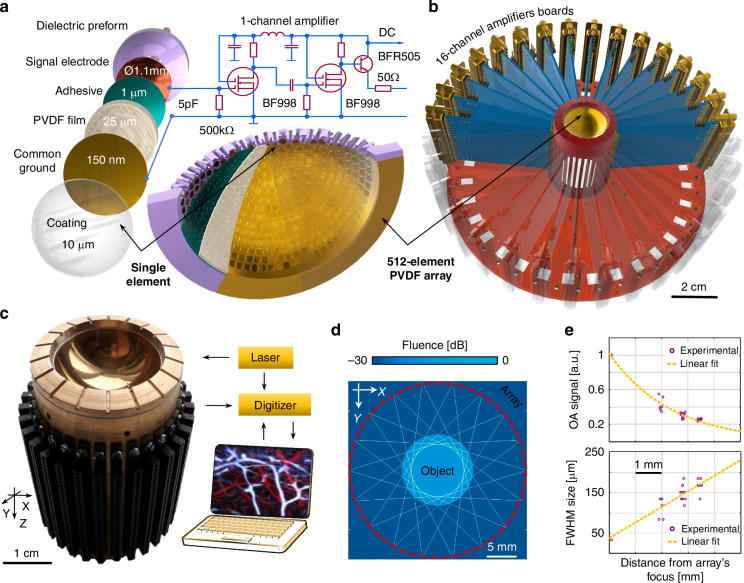
Concept of the ultrawideband 512-channel PVDF-based spherical matrix array for five-dimensional optoacoustic tomography. **a** Layout of the active detection surface and schematic of the tightly integrated pre-amplification circuitry; **b** Geometrical arrangement of the multi-channel electronics; **c** Schematic of the entire OA imaging system showing the circular arrangement of the illumination bundle containing 19 individual 0.7 mm diameter fibers with NA = 0.2. **d** Numerical simulation of the fluence distribution on the surface of 5 mm diameter sample, assuming a divergent Gaussian distribution of light emitted by each fiber. **e** Spatial resolution characterization of the OA imaging system obtained by translating an absorbing microsphere across the imaged FOV and measuring its reconstructed size and OA signal intensity

The spherical PVDF array was manufactured from a custom-made 15.02 mm radius spherical dielectric preform (Fig. 1a, see methods for a detailed description of the manufacturing process). It features 512 individual ~0.95 mm^2^ area apertures to which circular-shaped electrodes were attached. A 25 µm thick PVDF film (Precision Acoustics, UK) was bent to match the spherical shape of the preform and glued onto its surface, thus forming a spherical surface covering an angle of 135° (1.23π solid angle, 0.92 numerical aperture). A metallic electrode film (50 nm chrome and 100 nm gold) was sputtered on top of the concave surface of the PVDF film to form the ground electrode (Fig. 1a).

The weak OA signals collected by the relatively small elements (~2 pF capacitance) of the PVDF array were coupled to a 512-channel 30 dB preamplifier based on N-channel dual-gate MOS-FET transistors (BF998, NXP Phillips), which was realized with 32 double-sided boards each hosting 16 channels (Fig. 1b, see also Supplementary Information for a detailed description of the preamplifier). Low-frequency selection was performed with an interstage high-pass 0.3 MHz RC filter to eliminate the pyroelectric signal generated in the PVDF by the back-scattered photons^32^. A constant gain of 30 dB was further provided in the frequency range up to 40 MHz by varying the resistors in the drain circuits of the field-effect transistors.

For sample illumination, a custom-made bundle consisting of 19 multi-mode fibers (CeramOptec, Germany) was attached to the PVDF array in a circular illumination configuration (Fig. 1c), each fiber having 0.77 mm diameter and 0.22 numerical aperture (NA). All fibers were directed towards the center of the spherical array geometry to provide a uniform illumination over 5 mm diameter sample surface, which was confirmed with finite-element numerical simulations assuming a divergent Gaussian light distribution from each fiber (Fig. 1d). A short-pulsed (<10 ns) optical parametric oscillator (OPO) laser (Innolas Laser GmbH, Krailling, Germany) with wavelength tunable in the 660–1250 nm range on a per pulse basis and operating at a pulse repetition frequency (PRF) of 10 Hz was coupled to the bundle for OA signal excitation. A custom-made data acquisition system (DAQ, Falkenstein Mikrosysteme GmbH, Taufkirchen, Germany) was specifically designed to simultaneously digitize 512 signals at a sampling frequency of 100 MSPS (40 MHz effective bandwidth after pre-amplification), thus covering the full bandwidth of the transducer according to the Nyquist criterion.

### Array performance characterization

The PVDF array was carefully characterized to facilitate accurate tomographic imaging. The noise equivalent pressure (NEP) of PVDF array elements was further determined at 1 mPa/√Hz using calibrated hydrophone measurements (Model NH0500, Precision Acoustics Ltd, Dorchester, UK), which corresponds to 5.3 Pa over the useable 0.3–30 MHz bandwidth (see Supplementary Information). This is higher than the theoretically estimated NEP of 2 Pa, which is approximately equivalent to that of a piezocomposite transducer with a λ/4 matching layer for full-bandwidth detection (see Supplementary Information). The higher experimental NEP is in part due to the degradation of piezoelectric properties when the film is bent into a spherical surface. Note that, in comparison with PZT-based composite materials, PVDF films exhibit stronger induced electric field per unit stress (g33) hence also having higher theoretical sensitivity (see Supplementary Information).

Next, we mapped the exact locations of all the array elements. For this, a 20 μm diameter polyethylene microsphere (Cospheric BKPMS 20–27 µm) was attached to the tip of an optical fiber (Thorlabs UM22-100) using low-viscosity transparent cyanoacrylate glue and raster-scanned for a grid of 3 × 3 × 3 points equally separated by 2 mm. The position of each element was determined by trilateration from the measured times of flight. The spatial resolution was then estimated by imaging a Ø = 20 μm diameter microsphere embedded in an agar-based phantom, a common procedure in tomographic OA imaging^33^. The reconstructed FWHM of the microsphere was 40 μm and 30 μm in the lateral and axial directions, respectively. This corresponds to an effective spatial resolution of 35 μm (lateral) and 22 μm (axial) estimated as the square difference between the measured FWHM and the actual diameter of the microsphere, i.e., $\sqrt{{{FWHM}}^{2}-{{{\varnothing}}}^{2}}$. The phantom was subsequently translated along different directions and the reconstructed microsphere size and signal intensity were measured in each location to assess the effective FOV of the imaging system (Fig. 1e).

**Fig. 2:**
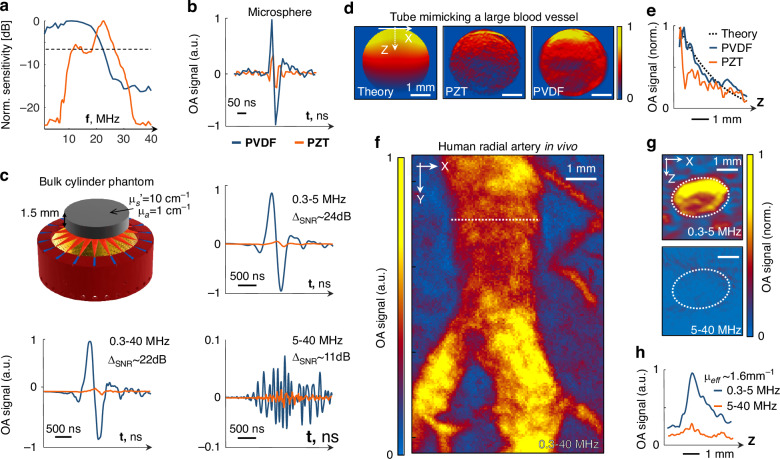
Direct comparison between the newly developed PVDF- and conventional piezocomposite (PZT)-based spherical arrays^34^ with similar geometry, number of elements and frequency range. **a** Normalized spectral sensitivity of the individual PVDF versus PZT array elements, as experimentally measured by etalon comparison using a calibrated needle hydrophone. **b** The PVDF array attains a higher SNR when recording OA responses from a 20 µm-diameter absorbing microsphere. **c** Imaging of a large uniformly illuminated bulk cylindrical phantom comprising a 1.5 mm thick 10 mm diameter scattering and absorbing agar slab. The PVDF attains an order of magnitude better SNR for low frequencies, which remains significantly higher in the medium frequency range. **d** When imaging a 3 mm diameter tube (mimicking a large blood vessel) placed along the Y axis, the PVDF-based detection results in slow (theoretically anticipated) exponentially decaying responses whereas the PZT array fails to detect the expected OA waveforms, primarily due to lack of sensitivity in the lower frequency band. **e** Simulated versus experimental signal profiles through the vessel demonstrating the good correspondence between the theory and experimental results obtained with the PVDF array. **f** In vivo imaging of a human radial artery further demonstrates the importance of ultrawideband detection. **g** In the X-Z projection of the volumetric image, the low frequency OA signal content (0.3–5 MHz) clearly reveals the smooth light fluence decay across the artery in the depth (Z) direction whereas the large artery is undetectable when performing image reconstruction with the high frequency signal components (5–40 MHz). **h** The corresponding low frequency signal profile through the artery corresponds to an effective optical attenuation coefficient of ~1.6 mm^-1^ (assuming an exponential decay), closely resembling the known blood optical parameters at the 850 nm illumination wavelength

We then directly compared the detection performance of the PVDF array to a previously reported high-frequency piezo-composite (PZT) spherical array having similar characteristics, namely, 510 individual detection elements, 1 mm element diameter and 15–35 MHz usable detection bandwidth^34^. An ultrawideband spectral sensitivity of the PVDF was experimentally determined using calibrated hydrophone measurements, in stark contrast to the relatively narrowband (<80% bandwidth) response of the PZT lacking sensitivity in the low frequency band (Fig. 2a), which is in congruence with previous reports^35^. In the high frequency 25–40 MHz range, mostly suitable for visualizing objects tens of micrometers in size, such as 20 µm-diameter absorbing microsphere (Fig. 2b), the spectral response of the PVDF sensors approximately matches the PZT sensors yet providing higher detection sensitivity. Moreover, when imaging large scattering and absorbing objects (Fig. 2c), the PVDF detector provides more than an order of magnitude better sensitivity to low frequencies (0.3-5 MHz, ~24 dB SNR increase), which remains superior also in the medium frequency range (5-25 MHz, ~11 dB SNR increase). Importance of the low frequency components of OA signals stems from the fact that quantitative imaging can only be achieved if the smooth spatial variations in light fluence associated to optical attenuation in diffuse tissues can accurately be captured^36^. This can be appreciated by comparing the OA images rendered with the PVDF versus PZT array from a cylindrical tube mimicking a relatively large 3 mm diameter blood vessel (Fig. 2d). While images rendered with the PZT array mainly emphasize boundaries of the vessel, the PVDF array is also capable of capturing the smooth signal decay with depth across the vessel, matching well the theoretical finite-element simulations based on light diffusion equation in turbid media (Fig. 2e).

The enhanced performance of the PVDF array for imaging large absorbing structures is also evinced from the OA images of the radial artery in a human wrist (Fig. 2f). When closely inspecting the cross-section (X-Z projection) of the 3D image volume (Fig. 2g), the power spectral density of the OA signal from the radial artery is mainly concentrated in the low 0.3–5 MHz range (Fig. 2h), which could only be captured owing to the ultrawideband detection bandwidth of PVDF. The higher frequency range between 5-40 MHz is thus shown to be poorly suited for OA imaging of large (1–3 mm diameter) vessels. In principle, the vessel contours can be also resolved with high-frequency PZT transducers and visualized in maximum intensity projections (MIPs)^37^. However, the reconstruction precision across multiple spatial scales has important implications when it comes to quantifying functional and molecular information based on OA images^38–40^. The ability to accurately record signals over full bandwidth, including low frequency components, further allows accurately capturing decay of the light fluence as it propagates through the radial artery. By considering the full signal bandwidth, it can readily be determined that the light propagation across the artery corresponds to an effective optical attenuation coefficient of ~1.6 mm^-1^ (assuming an exponential decay), closely resembling the known blood optical parameters at the 850 nm illumination wavelength^41^.

### Real-time volumetric human micro-angiography

**Fig. 3:**
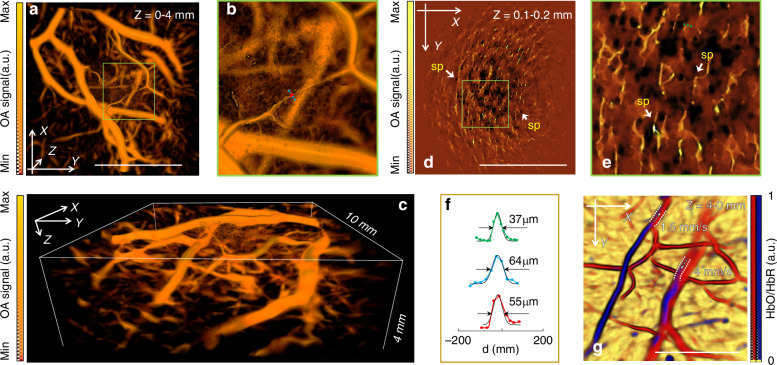
Single‐shot volumetric optoacoustic reconstructions of the human palm vasculature, acquired without any spatial or temporal averaging. **a** Compounded volumetric rendering of deep vessels (depth range 0–4 mm) in top view. **b** Close‐up view. **c** Side view. **d** Volumetric renderings of the superficial vascular plexus (depth range 100–200 µm) revealing multiple sweat pores (sp). Top view maximum intensity projection is shown. **e** Close-up view. **f** Representative OA signal line profiles measured across both small superficial capillaries and larger deep-seated vessels. **g** Multispectral volumetric rendering of relative oxygenation ratio (HbO/HbR) within the venules. Repeated volumetric optoacoustic acquisitions of blood oxygenation patterns (Supplementary Video 2) can be used to estimate slow venous blood flow velocities on the order of a few millimeters per second. All scale bars are 5 mm. Functional and real‐time angiographic performance of the array can be viewed in Supplementary Videos 1–2

We subsequently acquired time-lapse 3D data by single pulse laser probing of the human palm (no signal averaging). Owing to its ultrawide bandwidth, the system is capable of volumetric rendering of larger (>1 mm diameter) and deeper palm vessels (Fig. 3a–c), while simultaneously capturing small (30–50 µm) vessels and other structures located in the superficial skin layers at 100–200 µm depth (Fig. 3d, e). Dark round spots of about 100 µm in size are sweat gland ducts normally having a distribution density of ~250 pores/cm^2^^42^. Those were previously visualized by raster scan optoacoustic mesoscopy (RSOM) employing high-frequency single-element focused transducers^43^, which, however, required prolonged image acquisition times on the order of minutes. Also distinguishable are thin extended structures generating strong OA contrast that may be identified as vessels belonging to the superficial vascular plexus^26^. The maximum depth of the vessels observed in the images is ~3–4 mm (Fig. 3c). Microvasculature with diameter in the 55–75 µm range could clearly be resolved (Fig. 3b, e) with the smallest diameter of resolved vessels being 37 µm (Fig. 3f). Note that large vessels are generally difficult to visualize with high frequency PZT arrays, while their low frequency counterparts are unable to provide sufficient spatial resolution for microvascular imaging^44–48^. The broadband PVDF array thus uniquely enables imaging vasculature at multiple spatial scales, a crucial trait when it comes to quantitative characterization of the structure and function of vascular networks in health and disease.

The trade-off between achievable resolution and FOV (clearly visible in Fig. 3d) is consistent with what has been observed with PZT-based arrays of different central frequencies^49^. The relatively small FOV covered by the full bandwidth of the sensors has been expanded by combining multiple volumetric images acquired by slowly translating the sample and/or the array relative to each other. The image compounding has been facilitated by the large number of distinctive vascular features identified in the images. In this way, an extended 3D image volume covering 10 × 10 × 4 mm^3^ is rendered after stitching multiple individual volumes (Fig. 3a–c) via Fourier-based compounding of the adjacent volumetric frames, as previously described^50^. In this way, the high resolution in the center of the FOV can be extended over larger areas so that more microvessels become visible. Note that the effective FOV of the array, along with light attenuation, also determines the achievable imaging depth. The individual frames acquired during this scan are shown in a movie available in the on-line version of the journal (Supplementary Video 1), showcasing the real-time 3D imaging performance of the system.

We further demonstrate that repeated real-time volumetric optoacoustic acquisitions of the blood oxygen saturation (sO_2_) transients can be used to estimate slow venous blood flow on the order of a few mm/s (Fig. 3g and Supplementary Video 2). This is achieved by analyzing five-dimensional (5D, time-lapse spectroscopic volumetric) OA data acquired at multiple frames per second and tracking motion of the sO_2_ patterns between consecutive frames. While conventional OA imaging cannot provide Doppler-based measurements at such slow flow speeds, the fast spectroscopic changes captured with high temporal resolution by our system enable inferring bulk flow speeds in vessels of different sizes in real time.

### Non-invasive imaging of cerebral oxygenation changes in a mouse brain

**Fig. 4:**
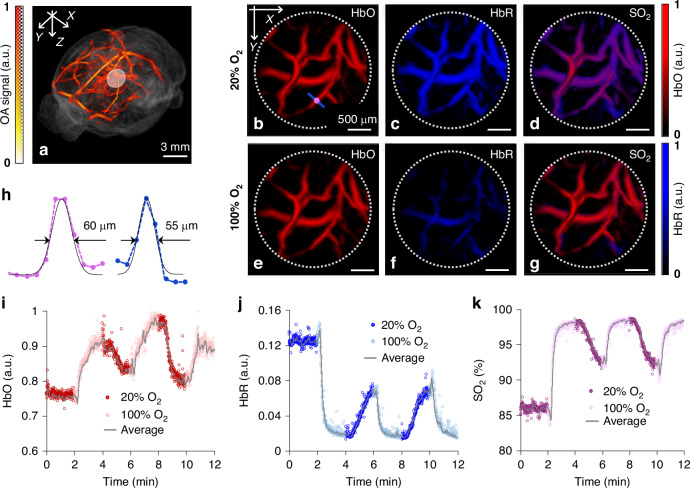
Noninvasive (through intact scalp and skull) OA imaging of blood oxygen saturation changes in mouse cerebral vessels in response to varying oxygen levels in the breathing gas mixture. **a** Compounded large-scale volumetric OA image of the mouse brain acquired at 850 nm superimposed onto an MRI-based mouse brain atlas; **b**–**g** Snapshots of the representative distributions of the spectrally unmixed HbO and HbR components in selected region (labeled in **a**) during the normoxic (20% O_2_) and hyperoxic (100% O_2_) phases of the experiment; **h** Cerebral vasculature down to 55 µm could be clearly resolved in these transcranial imaging experiments (the corresponding cross-section is labeled in **b**). **i**–**k** Dynamics of HbR, HbO and sO_2_ levels averaged over 2.5 × 2.5 × 0.7 mm^3^ volume. The volumetric spatio-temporal dynamics of the sO_2_ changes are best visualized in Supplementary Video 3

The 5D (spectroscopic, time-resolved, volumetric) performance of the system was subsequently demonstrated by noninvasive visualization of oxygen saturation changes in the murine cerebral vasculature induced with a breathing gas stimulation paradigm^51^. In particular, the breathing gas was switched between hyperoxic (100% O_2_) and normoxic (medical air, 20% O_2_) conditions every 2 min. We subsequently acquired a large-scale structural volumetric OA image of the mouse brain at 850 nm wavelength by means of the 3D image compounding^50^ and manually registered it onto an MRI-based Allen mouse brain atlas^52^ using major known vascular features (Fig. 4a). We further acquired real-time multi-spectral (5D) data from a selected FOV (marked in Fig. 4a) during the breathing stimulation experiments at 700, 730, 760, 800 and 850 nm wavelengths by fast switching of the laser wavelength on a per-pulse basis. This resulted in 0.5 s time resolution between two consecutive volumetric multi-spectral datasets, which was sufficient for capturing cerebral hemodynamics changes. Representative snapshots of the spectrally unmixed distributions of oxy- (HbO) and deoxy-hemoglobin (HbR) in cerebral vessels are shown under normoxic and hyperoxic conditions (Fig. 4b–g). Cerebral vasculature down to 55 µm could be clearly resolved in these transcranial imaging experiments (Fig. 4h). The corresponding temporal variations of the blood oxygenation parameters (HbR, HbO and sO_2_), averaged over 2.5 × 2.5 × 0.7 mm^3^ volume are depicted in Fig. 4i–k. Relative changes of 10–15-% in the sO_2_ levels within cerebral vessels were observed during 20% and 100% oxygen breathing cycles. The dynamics of sO_2_ changes associated to synchronous multidirectional shifts in the levels of HbO and HbR was in agreement with previous reports^51,53^. The time-lapse sequence of the acquired spectroscopic images is also visualized in a movie available in the on-line version of the journal (Supplementary Video 3).

### Whole-brain imaging of hemodynamic response to peripheral sensory stimulation

**Fig. 5:**
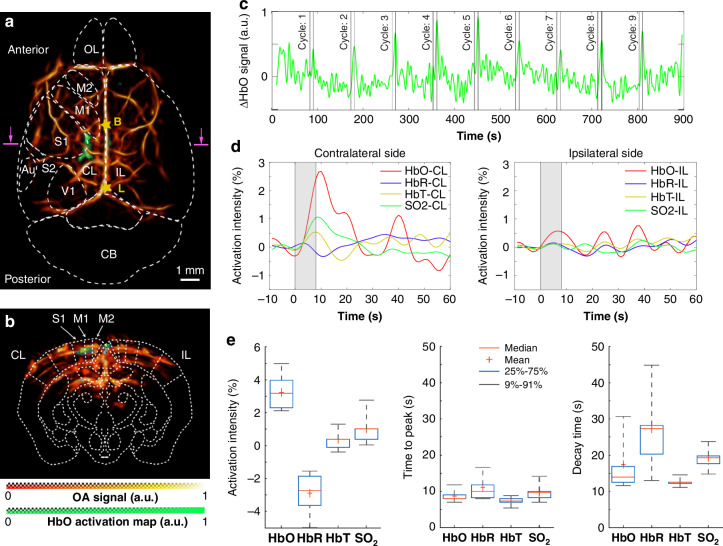
Noninvasive functional OA imaging of mouse brain hemodynamic responses upon electrical hindpaw stimulation. **a** Activation map of the HbO component labeled in green colormap overlaid to the structural large-scale OA image in top view; **b** Cross-sectional view of the HbO activation map depicted in a coronal plane. **c** Time-lapse traces of the HbO signal averaged across 0.3 × 0.3 × 0.1 mm^3^ volume of interest in the contralateral hemisphere, as indicated by the purple box in **a**, **b**; **d** time courses of HbO, HbR, HbT and sO_2_ hemodynamic components from both contralateral and ipsilateral regions, as indicated by the purple boxes in **a**, **b**. Averaged traces recorded from all the stimulation experiments are shown; **e** statistics of the activation intensity (left), time-to-peak (TTP, middle) and decay time (right). Au auditory cortex, Cb cerebellum, CL contralateral, IL ipsilateral, M1 primary motor cortex, M2 secondary motor cortex, OB olfactory bulb, S1 primary somatosensory cortex, S2 secondary somatosensory cortex, V1 primary visual cortex, B bregma, L lambda

We further demonstrate the application of the proposed system for whole-brain imaging of hemodynamic responses to peripheral sensory stimulation, which has been a subject of previous investigations^54^. A stimulation paradigm inspired by BOLD fMRI was adopted for this experiment^55^ with bipolar rectangular electrical pulses of 0.5 ms duration and 1.0 mA intensity applied to the left hindpaw at a stimulus frequency of 4 Hz with onset time of 8 s and a burst interval of 82 s. The 5D OA recordings were performed to map the changes in multiple hemodynamic parameters during and after the stimulation pulses. For localizing the brain activation, the anatomical high-resolution OA scan at the 850 nm wavelength was overlaid to the brain atlas^52^ (Fig. 5a). Figure 5c displays temporal dynamics of the unmixed HbO signal from 0.3 × 0.3 × 0.1 mm volume of interest (VOI) located at a depth of ~0.1 mm from the brain surface (cross-section labeled in pink in Fig. 5a). For functional data analysis, a custom processing pipeline inspired by statistical parametric mapping (SPM)^56^ was developed (see Methods) with the regressor obtained by convolving the stimulation paradigm with a modified SPM canonical hemodynamic response function (HRF). Activation map of HbO was calculated and overlaid to the single-wavelength (structural) OA volume, as shown in Fig. 5a, b. Note that all the active voxels shown in the activation map correspond to statistical significance of *p*-value < 0.05 with paired *t*-test. Localized activations were clearly observed from the primary somatosensory cortex (S1) as well as from primary motor cortex (M1) on the contralateral side while no obvious activation occurred on the ipsilateral side. When closely inspecting the averaged activation time courses of the various hemodynamic components, simultaneous increasing trends in HbO, HbT and sO_2_ and decreasing trend in HbR are observed in response to the electrical hindpaw stimulation (Fig. 5d, e). The HbO component exhibits the highest activation intensity (4.22 ± 1.10%), as compared to only 2.01 ± 0.91% for sO_2_, -1.90 ± 1.24% for HbR and 1.38 ± 0.61% for HbT. In contrast, the HbR holds the longest time-to-peak (TTP) value (11.11 ± 4.00 s) compared to 9.69 ± 2.19 s for sO_2_, 8.68 ± 1.70 s for HbO and 7.33 ± 1.02 s for HbT. The HbR component also has the longest decay time (27.28 ± 11.68 s) in comparison to sO_2_ (19.32 ± 3.60 s), HbO (17.45 ± 7.50 s) and HbT (12.57 ± 1.13 s).

## Discussion

The presented results demonstrate that the newly developed polymer-based (PVDF) high-density spherical matrix array can capture the full frequency bandwidth of the generated OA signals, thus facilitating accurate reconstructions of absorbers with dimensions spanning multiple spatial scales. OA is known to uniquely cover microscopic to macroscopic scales with the same optical-absorption-based contrast to render functional and molecular information from deep tissues. However, different spatial scales are encoded in different frequency components of the broadband OA signals excited with short-pulsed lasers. Hence, broadband detection is essential to fully exploit the multi-scale OA imaging capabilities. We have shown that the newly introduced PVDF-based array system is capable of accurately rendering boundaries and absorbing structures of different sizes while further enabling prediction of the light attenuation in deep tissues. The multi-scale imaging capacity was clearly observed in the angiographic images of the human palm, where microvascular structures branching out from other large vessels were resolved. We have also demonstrated real-time volumetric visualization of moving blood oxygenation patterns inside veins at speeds of only a few mm/s with high resolution and contrast, not attainable with other imaging modalities at the resolution - penetration scale provided by our system.

Given its ultrawideband detection ability, an obvious trade-off exists between resolution and FOV of the system with the smallest absorbing structures (highest resolving capacity) mainly observed in the central part of the imaged volume, while larger vessels could be resolved across a wider FOV. The relatively small FOV covered with the full transducer bandwidth is mainly related to the size of individual elements and their associated frequency-dependent directivity. This, along with light attenuation in deep tissues, establishes the achievable imaging depth of the system. The effective FOV can potentially be enlarged with an array consisting of a larger number of smaller sensors at the expense of a lower detection sensitivity. Alternatively, we have shown that the FOV can readily be expanded by scanning along the sample to the detriment of temporal resolution of the measurement^5^.

The large array bandwidth was further shown to facilitate high-resolution transcranial images of the mouse brain. Previously, real-time volumetric imaging of the murine cerebral vasculature has only been achieved with relatively narrowband PZT-based arrays having low central detection frequency in the 5 MHz range^10,29,49,57,58^. While higher frequencies may generally be affected by acoustic distortions of the skull, the recently reported detailed analysis of ultrasound transcranial propagation revealed frequency bands with relatively low skull acoustic insertion loss^59,60^, which may facilitate transcranial OA imaging with superior spatial resolution. The achievable resolution in vivo is also affected by speed of sound heterogeneities (e.g. in the skin) as well as by acoustic distortion and attenuation of high-frequency components in soft tissues^61,62^. In contrast to other whole-brain functional neuroimaging techniques, such as blood-oxygen-level-dependent (BOLD) fMRI^63,64^ and functional ultrasound (fUS)^65,66^ that are primarily sensitive to blood volume and flow, we were able to simultaneously map multiple hemodynamic parameters (HbO, HbR, HbT and sO_2_) with high spatial and temporal resolution using the OA method, which is crucial for the understanding of neurovascular and neurometabolic coupling mechanisms and the related diseases^67–69^. Recent studies have also shown the capacity of directly observing fast calcium activity in the mouse brain with OA^9,49^.

High-density piezo-composite arrays on a curved spherical geometry are known to be ideally suited for high-performance volumetric OA imaging^9,10^. However, manufacture of such arrays is hampered by the need to bend the piezo-composite material while attaching suitable matching layers with a high level of precision. This is particularly challenging for the thin layers of high-frequency arrays aimed at high resolution imaging. Compared to PZT materials, thin PVDF foils provide a broader detection bandwidth and better acoustic impedance matching to surrounding water. Thereby, higher sensitivity is expected for most frequencies despite the fact that the electromechanical coupling coefficient is lower for PVDF than for PZT^70^. Yet, given the broad bandwidth and high electrical impedance of PVDF, careful design of the pre-amplification circuitry is crucial for avoiding signal attenuation and bandwidth contraction due to capacitive loading. Importantly, the array introduced in this work exhibited superior sensitivity for low frequency (<5 MHz) components, which is essential for quantification of OA images of optically diffuse tissues.

High-frequency spherical arrays are also challenging to realize with alternative technologies, such as CMUT- or optical-based ultrasound detectors, due to various technical limitations, such as lack of mechanical flexibility, sensitivity and/or parallelization capacity. Moreover, the mechanical flexibility and stability of PVDF-based arrays can be used to expedite development of unconventional OA illumination and detection configurations optimized for specific applications. For instance, spherical arrays featuring apertures for light delivery have been used in hand-held scanners providing superb 3D angiographic imaging performance^44,71^. Hand-held probes based on concave or multi-segment arrays of cylindrically-focused transducers have also been shown to clearly outperform standard linear array probes^57^ by enhancing visibility of arbitrarily-oriented blood vessels in the reconstructed OA images^10^. Curved (concave) arrays are also often employed in whole-body small animal OA scanners to mitigate limited-view effects^72–74^ and increase visibility of vasculature and organs^75,76^. Finally, the flexibility of PVDF films can potentially be exploited for designing advanced OA endoscopic imaging systems^77^ or other miniaturized and wearable devices e.g. to study free behavior^29^.

All in all, the powerful ultrawideband high density arrayed detection capabilities introduced in this work are poised to have a fundamental impact on the modus of interpretation of OA data and the ongoing clinical translation of OA modality. The multi-scale angiographic imaging capacity of PVDF arrays facilitates more accurate identification of key morphological and functional indicators representative of conditions that involve vascular alterations, such as diabetes^78^, cardiovascular diseases^79–81^ or cancer^40,77^. The newly introduced ultrawideband polymer-based spherical matrix array technology thus leverages the true potential of OA imaging for high resolution visualization of biodynamics across scales.

## Materials and methods

### Manufacture of the high-density PVDF spherical array

For assembling the multi-element array, hemispherical dielectric preform (15.02 mm radius, 0.92 numerical aperture, 512 cavities for electrodes) was manufactured with 10 µm precision using a 3D printer ProJet 3500 HDMax (3D Systems, USA). For accurate reproduction of its spherical shape and compliance of the hole sizes, multi jet printing process was based on VisiJet Crystal photo polymer and VisiJet S300 auxiliary supporting material. Subsequently, the construction was cleaned using a special support removal procedure. The 512-element copper electrodes (0.95 mm^2^ effective area) were then manufactured with 1 µm precision using turning lathe Slantbed-Microturn 100 CNC (HEMBRUG, Netherlands). Positions of the array elements were determined with a sputtering mask defining the signal electrodes, while the common ground electrode for all elements was attached to the opposite side of the PVDF layer. The central positions of the individual detectors were equidistant over both elevational and azimuthal angles of the spherical coordinate system. After soldering individual 0.27 mm diameter micro-coaxial wires (44 AWG, Alpha Wire), the signal electrodes were mounted onto the 512 cavities of the dielectric preform. Rigid fixation of the electrodes and the signal wires was secured by custom-made epoxy-based compound with ~4 MRayl acoustical impedance, effectively matching acoustical impedance of the PVDF film to avoid undesired reverberations. The 25 µm thick PVDF film (Precision Acoustics, UK) was stretched using a steel ball to attain a hemispherical shape. The film was then glued with a custom-made adhesive to the inner surface of the dielectric preform. A metallic electrode film (50 nm chrome and 100 nm gold) was layered on top of the concave detection surface using custom-made low-temperature (80° C) vacuum sputtering machine to form the ground electrode (Fig. 1a). For protection and hydro isolation, ~10 µm layer of custom-made adhesive with ~4 MRayl acoustical impedance was placed on top of the ground electrode.

### Pre-amplification electronics

To ensure optimal detection bandwidth and sensitivity, 512 matching amplification channels were designed to be located in the closest possible proximity to the individual array elements (Fig. 1b). Due to its excellent noise performance and low cost, a field-effect double-gate transistor was selected as an input stage of the amplifier. Since single stage had insufficient voltage gain, a two-stage amplification circuit and an emitter follower were used to match the amplifier with a 50 Ohm link. The amplifier was powered by a unipolar 5 V source. The preamplification architecture based on field-effect double-gate transistors made it possible to maximize frequency bandwidth of the PVDF detectors. After connecting the 512 impedance-matching pre-amplification channels to the acoustical head, the circuitry was placed into a shielded casing which contained 19 grooves for embedding output ends of the custom-made fiber bundle (Fig. 1c). To cope with the high heat generation of the 512-channel amplifier consuming ~50 W of electric power, a cooling system was designed consisting of an immersion chamber, a forced air ventilation system, and a forced shutdown system for the amplifiers. The system of controlled air ventilation was tuned to maintain the temperature in the immersion chamber at 30 °C, whereas the forced shutdown system of the amplifiers was set to operate when the temperature in the immersion chamber reaches 45 °C, thus ensuring a safe margin from the PVDF depolarization threshold of 80 °C.

### Optoacoustic signal acquisition, image reconstruction and processing

Parallel digitization of the detected OA signals was accomplished with a custom-made high-speed DAQ (Falkenstein Mikrosysteme GmbH, Taufkirchen, Germany). It consists of 16 acquisition cards (32 channels each) providing 12 bits vertical digital resolution, 100 MHz sampling frequency and 40 MHz input frequency band. Synchronization was achieved by triggering acquisition with the Q-switch output of the pulsed laser source (Innolas GmbH, Krailling, Germany). It generates <10 ns duration pulses with 10 Hz repetition rate and wavelength tunable in the 660-1250 nm range.

For each laser pulse, a 3D image was tomographically reconstructed with an efficient 3D model-based algorithm, which has previously been shown to provide superior reconstruction performance, particularly in the context of spectroscopic optoacoustic measurements^82^. Prior to image reconstruction, the OA signals were band-pass filtered with cut-off frequencies between 0.3 and 40 MHz. A fine voxel size of 1 × 1 × 1 µm^3^ was chosen for the precise resolution characterization experiments. For rest of the images, the voxel size was set to 25 × 25 × 25 µm^3^. The vascular images were enhanced by applying a mask that was generated by using 3D Frangi vesselness filter^83^ at 25–400 µm spatial scales. The signals acquired at multiple wavelengths were further processed to spectrally un-mix the HbO and HbR bio-distributions. For this, the signals were first normalized by the corresponding signals recorded from an ink phantom with a calibrated absorption spectrum in order to correct for wavelength dependence of the laser energy. Un-mixing was then performed in the signal domain via spectral fitting^40^ to the known absorption spectra of HbO and HbR adopted from literature^84^. Tomographic image reconstruction of the HbO and HbR bio-distributions was performed considering the un-mixed signal components. The binary mask for the HbO and HbR distributions was obtained by applying 25–100 µm 3D Frangi vesselness filter to the unmixed HbT distribution.

### Array calibration, resolution and sensitivity characterization

The precise location of the array elements in 3D space was mapped via trilateration of OA responses obtained from a point source absorber. For this, a ~24 μm diameter polyethylene microsphere (Cospheric BKPMS 20–27 µm) was attached to the tip of an optical fiber (Thorlabs UM22-100) and raster-scanned for a grid of 3 × 3 × 3 points equally separated by 2 mm with the central point placed in the antenna focus. The algorithm used for trilateration comprised of searching for the optimal coordinates of the individual array elements on a spherical surface by minimizing the residual. It used as inputs the measured times-of-flight for the different positions on the sphere. The spatial resolution was estimated by imaging a ~20 μm microsphere embedded in an agar-based phantom along with the output ferrule of a fiber bundle consisting of 400 individual fibers (CeramOptec GmbH, Bonn, Germany). The distance between the output of the fiber bundle and the microsphere was ~3 mm. The sphere was positioned at the spherical center of the array so that the generated OA signals could be efficiently collected with all the array elements. The spatial resolution was estimated as the full width at half maximum (FWHM) of the reconstructed spheres for a single frame (no averaging), which represents a good estimate of the expected resolution for the in vivo imaging experiments. During the characterization measurements, the laser wavelength was tuned to 720 nm while the spherical array was oriented upwards with the medium surrounding the microspheres filled with pure water.

### Phantom experiments

Phantom experiments were performed to compare the effective bandwidth, sensitivity and quantitative imaging performance of the newly developed PVDF array with those of a previously reported PZT-based spherical array featuring similar dimensions and central frequency^34^. Specifically, the latter consisted of 510 elements with 1 mm diameter arranged over a 15 mm radius hemispherical surface covering 180° angle (2π solid angle). First, a ~ 24 μm polyethylene microsphere (Cospheric BKPMS 20–27 µm) was embedded in an agar phantom and imaged with both arrays at 720 nm wavelength. The phantom was positioned in a custom-made holder featuring an aperture to introduce the output of a fiber bundle guiding the light beam. In this way, the optical fluence at the particle location was kept constant for different acquisitions. OA signals were acquired with both arrays after positioning the microsphere at the center of their spherical surfaces with manual positioning stages. Single-shot acquisitions (no averaging) were used for comparing the signal SNR, while 200 averages were applied to determine the effective detection bandwidth.

The second phantom experiment concerned imaging of a large uniformly illuminated tissue-mimicking bulk cylindrical phantom comprising a 1.5 mm thick 10 mm diameter agar slab. The agar was mixed with ink and intralipid to achieve absorption coefficient of *µ*_a_ = 1 cm^-1^ and reduced scattering coefficient of *µ*_s_’ = 10 cm^-1^. Another agar phantom was prepared that contained a 3 mm cylindrical tube to mimic a large blood vessel. A mixture of ink and intralipid was used to achieve absorption coefficient of *µ*_a_ = 4.3 cm^-1^ and reduced scattering coefficient of *µ*_s_’ = 16 cm^-1^, corresponding to optical properties of blood at 800 nm^84^. In this case, the laser energy was delivered from the top by means of a different fiber bundle having 3 outputs with equal angular separation of 45^o^. Imaging was performed with both PVDF and PZT arrays with the illumination fixed in the same position relative to the phantom. Signal averaging for 500 consecutive laser pulses was performed.

### Numerical simulations

Finite-element numerical simulations were performed to verify accuracy of the fluence distribution map in the cylindrical tube phantom (placed along Y axis) reconstructed with the PVDF array (Fig. 2d, e). The simulations were performed in COMSOL 5.0 (Comsol Inc., Burlington, MA, USA) by considering the Helmholtz equation function (hzeq) as a partial differential equation (PDE) in a two-dimensional geometry corresponding to the cylindrical cross-section of the phantom. The diffusion coefficient was calculated via *D* = 3(*μ*_a_ + *μ*_s_’)^-1^ and the source term f was set to 0 within the cylindrical volume. A number of 25 equally spaced point sources (source term *f* = 1) were placed at 0.1 mm distance from the cylindrical surface covering a total angle of 120° to mimic the illumination profile in the X-Z plane in the experimental set-up. A Robin boundary condition was used with the flux/source and absorption/impedance terms set to *g* = 0 and *q* = 1/2 A, respectively. The A constant was calculated to be 1.088 using the Keijzer approximation^85^ for a relative tissue/water refractive index of 1.034^84^. An extremely fine uniformly distributed mesh has been employed.

### Angiographic imaging of a healthy volunteer

Microvascular structures of the palm and wrist of a healthy volunteer (Caucasian skin) were imaged with the PVDF array. For this, the laser wavelength and PRF were set to 850 nm and 2 Hz, respectively. The energy density at the tissue surface was maintained below the 40 mJ/cm^2^ laser safety threshold for skin exposure at 850 nm^86^. For multispectral 5D imaging the PRF of the laser was set to 10 Hz and the wavelength was alternated between 700, 730, 760, 800 and 850 nm on a per-pulse basis for rapid acquisition of volumetric multi-spectral datasets (2 Hz spectroscopic volume rate). Acoustic coupling was guaranteed by filling the volume covered by the spherical array with deionized water. The palm of the volunteer was gently moved while maintaining direct contact with the water surface. Real-time preview was performed to localize the region of interest with a sequence of 100 volumetric frames subsequently acquired.

### Noninvasive imaging of murine cerebral hemodynamic changes

Real-time imaging of cerebral oxygenation changes in the mouse brain was performed fully non-invasively. For this, the head of an 8-week-old female athymic nude-Fox1nu mouse was placed in the focal area of the spherical array. To facilitate acoustic coupling, the space between the mouse and the active spherical detection surface was filled with water. A thin transparent polyethylene membrane isolated the mouse from the coupling medium with ultrasound gel further used to ensure good contact between the skin and the membrane. The PRF of the laser was set to 10 Hz and the wavelength was alternating between 700, 730, 760, 800 and 850 nm on a per-pulse basis for rapid acquisition of volumetric multi-spectral datasets. The experiment consisted in a breathing gas stimulation paradigm, where the inhaled gas was changed between normoxia (medical air, 20% O_2_) and hyperoxia (100% O_2_) for 2-min cycles and a total duration of 12 min. Physiologic parameters, including blood oxygenation, heart rate, and body temperature were continuously monitored throughout the experiments (PhysioSuite, Kent Scientific). The body temperature was kept around 37 °C with a heating pad. All experiments were performed in full compliance with relevant policies and institutional guidelines. Animal handling was performed in accordance with the Swiss Animal Protection Act and approved by the Cantonal Veterinary Office Zurich.

### Imaging brain responses to peripheral sensory stimulation

Imaging of brain hemodynamic responses to peripheral sensory stimulation was performed entirely non-invasively (scalp and skull intact) in 10 week-old female athymic nude-Fox1nu mice (*n* = 4). For induction, each mouse was intraperitoneally injected with a ketamine (100 mg/kg) and xylazine (10 mg/kg) anesthesia cocktail in a volume of 3 µl/g body weight. The bolus was separated into two smaller injections 5 min apart to prevent cardiovascular complications. During the experiment, oxygen/air mixture (0.2/0.8 L.min-1) was provided through a breathing mask. The same mouse positioning and OA acquisition parameters were used, as described in the previous section.

A typical BOLD fMRI stimulation paradigm was adopted for this experiment^55^. Briefly, bipolar rectangular electrical pulses of 5 ms duration and 1.0 mA intensity were applied to the left hindpaw at a stimulus frequency of 4 Hz with onset time of 8 s and a burst interval of 82 s using a stimulus isolator device (Model A365R, World Precision Instruments, USA). For each sequence, the complete pulse train duration was 900 s while the stimulus train was synchronized with the excitation light and data acquisition using an external trigger device (Pulse Pal V2, Sanworks, USA). After the experiment, mice were kept alive for future work.

A custom data analysis pipeline inspired by SPM12 (https://www.fil.ion.ucl.ac.uk/spm/)^56^ was developed for analyzing the spectrally unmixed hemodynamic signal components with the script written in MATLAB (2019b, MathWorks, USA). General linear model (GLM) was applied to a voxel-by-voxel based statistical analysis. Before the volumetric image data was fed into the statistical analysis model, motion estimation and correction were performed. Spatial smoothing (Gaussian kernel FWHM = 0.3 mm) was implemented and a high-pass filter with cut-on frequency of 1/135 Hz was used to detrend the time-lapse signal traces. In the GLM, the regressor was obtained by convolving the stimulation paradigm with a modified SPM canonical HRF (peaking at 2 s and returning to baseline at 5 s). The significance of the event-evoked responses in the observations was further evaluated with a contrast vector *c* = [1, 0]^T^; t statistical map (t-map) and probability levels (*p*-values) were calculated as the statistical inferences. Note that to render the 3D activation map, false discovery rate (FDR) controlling with *p*-values < 0.05 was introduced to the t-map. The activation map (i.e. FDR corrected t-map) together with the corresponding structural image reconstructed from single wavelength OA image acquired at 850 nm wavelength were manually overlaid to the Allen Mouse Brain Atlas 2015^52^ using Amira (version 5.4, Thermo Fisher Scientific, USA). Instead of using the predicted values from the GLM, time courses of the brain activation were obtained by retrieving raw signals from the corresponding 4D data (i.e. 3D volume time series). The calculated activation map served as the inference of VOIs including both contralateral and ipsilateral hemispheres. A time window including 10 s pre-stimulation, 8 s onset and 50 s post-stimulation was selected. Baseline signals of each stimulation cycle were calculated by averaging the 10 s pre-stimulation time series. The fractional signal changes were calculated subsequently and averaged across different stimulation cycles. To reveal the stimulus-evoked brain activation in a more robust way, all the trials acquired at slightly different anesthesia depth and from different mice were averaged.

## Supplementary information

Supplementary Video 1

Supplementary Video 2

Supplementary Video 3

Supplementary materials

## Data Availability

The data supporting the findings of this study are available from the corresponding authors upon reasonable request.
