# Supplementary material for: Ultrawideband high density polymer-based spherical array for real-time functional optoacoustic micro-angiography
Source: Light Sci Appl. 2025 Jul 7;14:239. doi: 10.1038/s41377-025-01894-y (PMC12234755; doi:10.1038/s41377-025-01894-y)
Supplement: Supplementary file 4 — Supplementary materials [file 41377_2025_1894_MOESM4_ESM.pdf]

Supplementary Information for

Ultrawideband high density polymer-based spherical array for real-time functional optoacoustic micro-angiography

Pavel V. Subochev[1,*,+], Xosé Luís Deán-Ben[2,3,+], Zhenyue Chen[2,3,4], Maxim B. Prudnikov[1], Vladimir A. Vorobev[1], Alexey A. Kurnikov[1], Anna G. Orlova[1], Anna S. Postnikova[1], Alexey V. Kharitonov[1], Mikhail D. Proyavin[1], Roman I. Ovsyannikov[1], Anatoly G. Sanin[1], Mikhail Y. Kirillin[1], Francisco Montero de Espinosa[5], Ilya V. Turchin[1], and Daniel Razansky[2,3,6 *]

[1]Institute of Applied Physics, Russian Academy of Sciences, Nizhny Novgorod, Russia

[2]Institute of Pharmacology and Toxicology and Institute for Biomedical Engineering, Faculty of Medicine, University of Zurich, Switzerland

[3]Institute for Biomedical Engineering, Department of Information Technology and Electrical Engineering, ETH Zurich, Switzerland

[4]Present affiliation: Institute of Precision Optical Engineering, School of Physics Science and Engineering, Tongji University, Shanghai, China

[5]Instituto de Tecnologías Físicas y de la Información – CSIC, Madrid, Spain

[6]Institute for Biological and Medical Imaging, Helmholtz Center Munich, Neuherberg, Germany

*E-mail: pavel.subochev@gmail.com; daniel.razansky@uzh.ch

+ - authors equally contributed to this manuscript

1. Theoretical comparison of the sensitivity of polyvinylidene difluoride versus piezocomposite materials

In the general case, piezoelectric transducers can be described through the equations of direct and inverse piezoelectric effect [1-3]:

$$\begin{matrix} E = \beta^S D - hS \\ T = -hD + c^D S \end{matrix} \quad or \quad \begin{matrix} D = \varepsilon^S E + eS \\ T = -eE + c^E S \end{matrix} \tag{1}$$

where $E[V/m]$, $D[C/m^2]$ – electric field strength and inductance, $T[N/m^2]$ – elastic stress, S – linear deformation, $\varepsilon^S[F/m]$, $\beta^S[m/F]$ – permittivity and impermeability at constant strain, $c^E[N/m^2]$, $c^D[N/m^2]$ – elasticity constant at constant electric field strength and inductance, $e[C/m^2]$ – piezoelectric constant, $h[V/m]$ – piezoelectric strain constant.

The most convenient way to model a longitudinally oscillating piezoelectric transducer is to describe it as an electromechanical hexapole [4] or quadrupole [5]. To compare the sensitivity of polyvinylidene difluoride versus piezocomposite materials, we will hereby use the theory of an

electromechanical quadrupole, which describes one-sided radiation or reception of a transducer in a simpler fashion. In this analysis of acoustic systems, electromechanical analogies are commonly used by representing electric voltage U via force F and electric current I via an oscillatory speed V [6]. The conversion of the mechanical energy of ultrasonic wave oscillations into electrical energy is described using the chain matrix of the converter with frequency-dependent complex coefficients A_{11}, A_{12}, A_{21}, A_{22}. In general, the matrix of the transducer in the modes of transmission and reception is expressed as follows

$$\begin{matrix} U_{in} = A_{11}F_{out} + A_{12}V_{out} \\ I_{in} = A_{21}F_{out} + A_{22}V_{out} \end{matrix}, \quad \begin{matrix} F_{in} = A_{22}U_{out} + A_{12}I_{out} \\ V_{in} = A_{21}U_{out} + A_{11}I_{out} \end{matrix}. \tag{2}$$

where U_{in}, I_{in} – input voltage and current applied to the piezoelectric plate, V_{out}, F_{out} – oscillation speed and force generated on the working surface of the transducer in radiation mode, V_{in}, F_{in} – oscillation speed and force generated on the working surface of the transducer in receive mode, U_{out} – voltage arising on the plates of the piezoelectric element in receive mode, I_{out} – current flowing through the electrical load of the piezoelectric receiver. In the case of a passive quadripole the condition of invertibility of the chain matrix must be satisfied, namely $(A_{11}A_{22} - A_{12}A_{21}) = 1$.

The matrix system for a piezoelectric receiver is greatly simplified when reducing it to a P-type circuit, i.e.,

$$\begin{matrix} F_{in} = A_{22}U_{out} \\ V_{in} = A_{21}U_{out} \end{matrix} \tag{3}$$

Also, when calculating sensitivity of a piezoelectric receiver, it must be considered that the pressure created on the working surface differs from the pressure of the incident wave. It is expressed via

$$p_t = p_{inc} + p_{ref}, \text{ or } p_t = p_{inc}\frac{2z_0}{z_0+z_2} \tag{4}$$

where p_t, p_{inc}, p_{ref} – pressure of the transmitted, incident and reflected waves, z_0, z_2 – acoustic resistance of the piezoelectric element and the working environment.

From expressions (2,3,4), the transfer coefficient K_{rad} and sensitivity K_{rec} can be calculated through coefficients of the converter matrix as

$$K_{rad} = \left|\frac{p_{out}}{U_{in}}\right| = \left|\frac{F_{out}}{S_0 \cdot U_{in}}\right| = \left|\frac{1}{A_{11}S_0 + A_{12}/z_2}\right| \tag{5}$$

$$K_{rec} = \left|\frac{U_{out}}{p_{inc}}\right| = \left|\frac{2z_0}{z_0 + z_2} \cdot \frac{U_{out}}{p_t}\right| = \left|\frac{2z_0}{z_0 + z_2} \cdot \frac{S_0 \cdot U_{out}}{F_{in}}\right| = \left|\frac{2S_0}{A_{21}z_2S_0 + A_{22}}\right| \tag{6}$$

where S_0 is the transducer surface area, $z_2 = \frac{F_{out}}{S_0 \cdot V_{out}}$, $z_0 = \frac{F_{in}}{S_0 \cdot V_{in}} = \frac{A_{22}}{S_0 \cdot A_{21}}$.

The matrix coefficients are theoretically obtained from the equation of motion for a piezoelectric plate and the equations of direct and inverse piezoelectric effects [5], i.e.,

$$\begin{aligned} A_{11} &= \frac{l}{k_t v^* \sqrt{\varepsilon^* \varepsilon_0 \rho} S_0}\left[\frac{k_t^2 f_0}{\pi f}(a_{11} + jb_{11}) + (b_{21} - ja_{21})\right], \\ A_{12} &= \frac{l z_0}{k_t v^* \sqrt{\varepsilon^* \varepsilon_0 \rho}}\left[\frac{k_t^2 f_0}{\pi f}(a_{12} + jb_{12}) + (b_{22} - ja_{22})\right], \\ A_{21} &= \frac{2\pi f C l}{k_t v^* \sqrt{\varepsilon^* \varepsilon_0 \rho} S_0}(a_{21} + jb_{21}), \\ A_{22} &= \frac{2\pi f C l z_0}{k_t v^* \sqrt{\varepsilon^* \varepsilon_0 \rho}}(a_{22} + jb_{22}). \end{aligned} \tag{7}$$

The following parameters are considered $C = \frac{\varepsilon^* \varepsilon_0 S_0}{l}$ – transducer capacitance, $f_0 = \frac{v}{2l}$ antiresonant frequency, ε_0 – dielectric constant in vacuum, $l, \rho, k_t, \varepsilon^*, v^*$ – thickness, density, electromechanical coupling coefficient, complex dielectric constant and complex sound velocity of the piezomaterial. Coefficients a_{mn}, b_{mn} $(m, n = 1{,}2)$ are expressed in the following form

$$
\begin{aligned}
a_{11} &= \frac{\mathrm{tg}\dfrac{\pi f}{2f_0}}{k_1^2 + \mathrm{tg}^2\dfrac{\pi f}{2f_0}} & b_{11} &= \frac{k_1}{k_1^2 + \mathrm{tg}^2\dfrac{\pi f}{2f_0}} \\
a_{12} &= -\frac{k_1\,\mathrm{tg}\dfrac{\pi f}{2f_0}}{k_1^2 + \mathrm{tg}^2\dfrac{\pi f}{2f_0}} & b_{12} &= \frac{k_1^2 + 2\mathrm{tg}^2\dfrac{\pi f}{2f_0}}{k_1^2 + \mathrm{tg}^2\dfrac{\pi f}{2f_0}} \\
a_{21} &= \frac{k_1\left(\mathrm{ctg}\dfrac{\pi f}{f_0} + \mathrm{tg}\dfrac{\pi f}{2f_0}\right)}{k_1^2 + \mathrm{tg}^2\dfrac{\pi f}{2f_0}} & b_{21} &= \frac{k_1^2 - \mathrm{ctg}\dfrac{\pi f}{f_0}\cdot \mathrm{tg}\dfrac{\pi f}{2f_0}}{k_1^2 + \mathrm{tg}^2\dfrac{\pi f}{2f_0}} \\
a_{22} &= \frac{k_1^2\mathrm{ctg}\dfrac{\pi f}{f_0} + \mathrm{tg}\dfrac{\pi f}{2f_0}}{k_1^2 + \mathrm{tg}^2\dfrac{\pi f}{2f_0}} & b_{22} &= \frac{k_1\left(1 - \mathrm{ctg}\dfrac{\pi f}{f_0}\cdot \mathrm{tg}\dfrac{\pi f}{2f_0}\right)}{k_1^2 + \mathrm{tg}^2\dfrac{\pi f}{2f_0}}
\end{aligned} \tag{8}
$$

Here $k_1 = \frac{z_1}{z_0}$ is the ratio of the specific complex impedances of the piezomaterial z_0 and damper z_1.

The complex dielectric constant ε^* and the elasticity constant c^{D*} can be expressed in terms of their real parts and dielectric tangents $\tan\delta_e$ and mechanical losses $\tan\delta_m$ as [7]

$$\varepsilon^* = \varepsilon(1 - j\mathrm{tg}\delta_e) \tag{9}$$

$$c^{D*} = c^D(1 + j\mathrm{tg}\delta_m) \tag{10}$$

The complex speed of sound v^* can also be expressed in terms of its real part and mechanical loss as

$$v^* = \sqrt{\frac{c^{D*}}{\rho}} = \sqrt{\frac{c^D(1 + j\mathrm{tg}\delta_m)}{\rho}} = v\sqrt{(1 + j\mathrm{tg}\delta_m)} \tag{11}$$

When using a matching layer between the ultrasonic transducer and the medium (typically water or biological tissues), as well as when considering the input active resistance R in the electrical circuit of the converter, the transfer coefficient and sensitivity are calculated through matrix coefficients $\tilde{A}_{mn}$ $(m, n = 1{,}2)$, expressed in terms of coefficients A_{mn} as the following [5]

$$\begin{vmatrix} \tilde{A}_{11} & \tilde{A}_{12} \\ \tilde{A}_{21} & \tilde{A}_{22} \end{vmatrix} = \begin{vmatrix} 1 & 0 \\ 1/R & 1 \end{vmatrix} \times \begin{vmatrix} A_{11} & A_{12} \\ A_{21} & A_{22} \end{vmatrix} \times \begin{vmatrix} P_{11} & P_{12} \\ P_{21} & P_{22} \end{vmatrix}, \tag{12}$$

or after the matrix multiplication in the following form

$$\begin{gathered}\tilde{A}_{11} = A_{11}P_{11} + A_{12}P_{21} \\ \tilde{A}_{12} = A_{11}P_{12} + A_{12}P_{22} \\ \tilde{A}_{21} = \left(\frac{A_{11}}{R} + A_{21}\right)P_{11} + \left(\frac{A_{12}}{R} + A_{22}\right)P_{21} \\ \tilde{A}_{21} = \left(\frac{A_{11}}{R} + A_{21}\right)P_{12} + \left(\frac{A_{12}}{R} + A_{22}\right)P_{22}\end{gathered} \tag{13}$$

where coefficients P_{mn} are defined as

$$\begin{gathered}P_{11} = \cos\left(2\pi\frac{\lambda_1}{\lambda_0}\frac{f}{f_0}\right) \\ P_{12} = jS_0z_3\sin\left(2\pi\frac{\lambda_1}{\lambda_0}\frac{f}{f_0}\right) \\ P_{21} = j\frac{\sin\left(2\pi\frac{\lambda_1}{\lambda_0}\frac{f}{f_0}\right)}{S_0z_3} \\ P_{22} = \cos\left(2\pi\frac{\lambda_1}{\lambda_0}\frac{f}{f_0}\right)\end{gathered} \tag{14}$$

where z_3 is the specific acoustic impedance of the matching layer, λ_0 is the wavelength in a piezomaterial at the antiresonant frequency f_0, and λ_1 is the wavelength in the matching layer at the antiresonant frequency f_0.

Based on the main characteristics of the piezo material, the piezoelectric coefficient d_{33}, the piezoelectric voltage constant g_{33} and the energy conversion coefficient $d_{33} \times g_{33}$ can be written as follows [3,5]

$$\begin{gathered}d_{33} = \frac{1}{v}\sqrt{\frac{\varepsilon\varepsilon_0}{\rho}}\frac{k_t}{(1-k_t^2)} \\ g_{33} = \frac{1}{v}\sqrt{\frac{1}{\varepsilon\varepsilon_0\rho}}k_t \\ d_{33} \times g_{33} = \frac{1}{v^2\rho}\frac{k_t^2}{(1-k_t^2)}\end{gathered} \tag{15}$$

The given coefficients characterize the emitting and receiving abilities of the detector. Materials with a higher d_{33} value have better emissivity K_{rad}. Such materials are lead zirconate titanate (PZT) or PZT-based piezocomposites. At the same time, a larger value of g_{33} provides a higher sensitivity K_{rec} of the piezoelectric material. High g_{33} modulus is intrinsic characteristics of polyvinylidene difluoride (PVDF). The calculated values of d_{33} and g_{33} for PVDF and PZT-based piezocomposite are shown in Table 1.

Material properties	PVDF	PZT-based 1–3 composite
Density $\boldsymbol{\rho}$, kg/m^3	1780	4190
Velocity $\boldsymbol{v}$, m/s	2300	3200
Thickness $\boldsymbol{l}$, μm	25	64
Dielectric constant $\boldsymbol{\varepsilon}$	13.5	3400
Electromechanical coupling coefficient $\boldsymbol{k}_t$	0.15	0.59
Dielectric loss $\boldsymbol{tan\,\delta_e}$	0.015	0.02
Mechanical loss $\boldsymbol{tan\,\delta_m}$	0.065	0.05
Active input impedance $\boldsymbol{R}$, Ω	$5 \cdot 10^5$	50
Capacitance of piezo disk $\boldsymbol{C_0}$, pF	4.5	450
Piezoelectric coefficient $\boldsymbol{d_{33}}, pC/N$	18	758
Piezoelectric voltage constant $\boldsymbol{g_{33}}, V \cdot m/N$	0.147	0.016
Energy conversion coefficient $\boldsymbol{d_{33}} \times \boldsymbol{g_{33}}, 10^{-12}\ m^2/N$	2.64	12.4

Table S1. Comparative properties of polyvinylidene difluoride (PVDF) and lead zirconate titanate (PZT)-based 1-3 composite piezoelectric materials.

Considering the mathematical model described above and the properties shown in Table S1, sensitivity of an ultrasound sensor based on a polyvinylidene difluoride (PVDF) film can theoretically be compared to that of a typical lead zirconate titanate (PZT)-based 1-3 composite (Figure S1A). A thickness of 25 μm was used to model the PVDF film. The thickness of the PZT-based composite material was determined considering an antiresonance frequency of 25 MHz and longitudinal speed of sound in this material. The sensitivities were calculated for transducers coupled with water having acoustic impedance $z_2 = 1.5$ MRayl. A backing of copper ($z_1 \approx 42$ ·MRayl) was considered for the PVDF sensor corresponding to the electrodes used to manufacture the spherical array. On the other hand, a typical epoxy backing ($z_1 \approx 2.2\,\mathrm{MRayl}$) was considered for the sensor based on a PZT composite. Input impedances of $50\ \Omega$ and $500\ \mathrm{k}\Omega$ were taken for the PZT-based composite and the PVDF material, respectively (Table S1). For the PZT-composite-based sensor, the calculations were performed both with an optional matching layer between the piezomaterial and the load medium ($z_3 = \sqrt{z_0 \cdot z_2}, \lambda_1 = \lambda_0/4$) and with no matching layer. The sensitivity of the PVDF sensor exhibits higher (10.1 µV/Pa maximum value) sensitivity than the PZT-based sensor (3.8 µV/Pa maximum value). Also important is the fact that the PVDF sensor has significantly larger -6 dB detection bandwidth (from 0.1 to 30 MHz) than the PZT-based sensor (from 14 to 33 MHz).

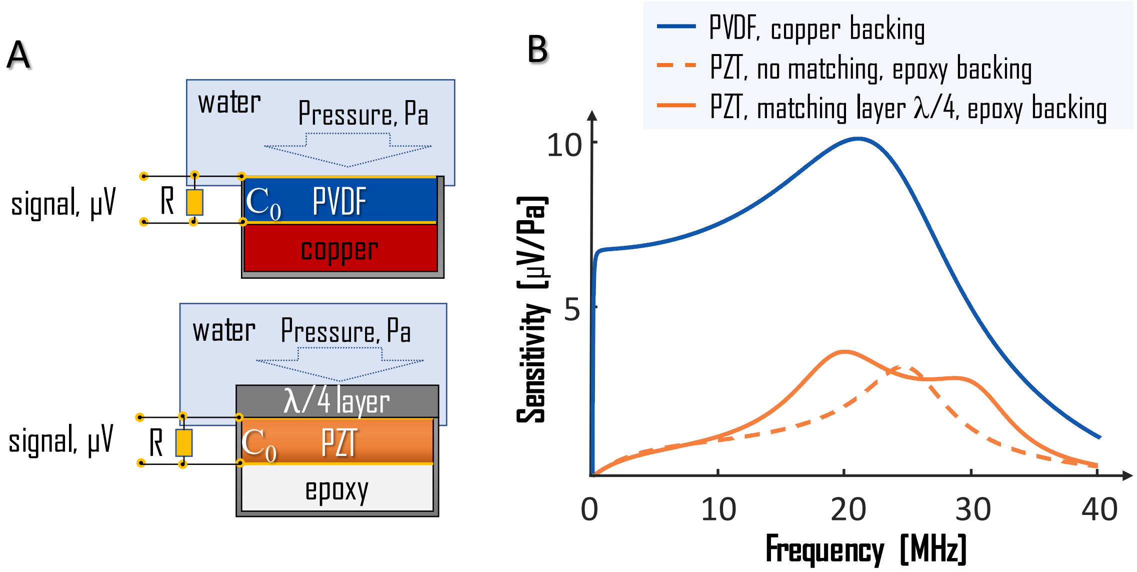

Figure S1. Sensitivity Analysis of PVDF and PZT-based detectors. (A) Mathematical model representation for both the Polyvinylidene difluoride (PVDF) sensor and the lead zirconate titanate (PZT)-based 1-3 composite sensor. (B) Theoretical plots illustrating sensor sensitivity as a function of frequency.

For estimating the theoretical thermal noise of the PZT-based composite and PVDF piezomaterials, we considered the known capacitance and the matching load resistances (Table S1). For room temperature and megahertz frequencies, the Nyquist formula can be used to estimate the thermal noise of an electrical circuit [8], i.e.

$$\langle U^2 \rangle = 4k_B T \int_{f_1}^{f_2} Re[Z(f)]df \qquad (16)$$

Considering the complex electrical impedance of the piezoelectric element switching RC circuit (Fig. S1A)

$$Z(f) = \frac{R}{1 + (2\pi f C_0 R)^2} - j\frac{2\pi f C_0 R^2}{1 + (2\pi f C_0 R)^2}, \qquad (17)$$

and performing the integration of Eq. (16), one obtains the following estimate for the thermal noise of the electrical circuit under consideration:

$$\langle U^2 \rangle_{[f_1, f_2]} = \frac{k_B T}{C_0}\frac{2}{\pi}\mathrm{atan}[2\pi R C_0 f]\Big|_{f_1}^{f_2}. \qquad (18)$$

With an infinitely wide reception frequency band $\frac{2}{\pi}\mathrm{atan}[2\pi R C_0 f]\Big|_0^{\infty} \sim 1$, Eq. (18) corresponds to the standard value for kTC-noise [9].

The noise equivalent pressure (NEP) is then calculated by considering the pressure noise averaged over the receiving frequency band, which can theoretically be estimated from (6) and (18) as follows

$$\langle NEP \rangle_{[f_1, f_2]} = \sqrt{\langle U^2 \rangle_{[f_1, f_2]}} / \langle K_{rec} \rangle_{[f_1, f_2]} \qquad (19)$$

Table S2 shows theoretical estimates of $\langle NEP\rangle_{[f_1,f_2]}$ calculated for PZT-based composite and PVDF piezomaterials at various receiving frequency ranges relevant for biomedical optoacoustic imaging applications. PVDF exhibits several folds better $\langle NEP\rangle_{[f_1,f_2]}$ for the frequency bands of 0.3-7 MHz and 7-40 MHz, although comparable value of $\langle NEP\rangle_{[f_1,f_2]}$ was estimated for the full bandwidth of 0.3-40 MHz.

	PVDF			PZT-based 1-3 composite with λ/4		
**Cutoff frequency low f_1, MHz**	0.3	0.3	7	0.3	0.3	7
**Cutoff frequency high f_2, MHz**	7	40		7	40	
**Thermal noise $\sqrt{\langle U^2\rangle_{[f_1,f_2]}}$, μV**	12.4	13.2	4.5	2.1	2.8	1.9
**Sensitivity $\langle K_{rec}\rangle_{[f_1,f_2]}$, $\mu V/Pa$**	6.9	6.7	6.6	0.6	1.9	2.2
**Averaged NEP $\langle NEP\rangle_{[f_1,f_2]}$, Pa**	1.8	2	0.7	3.5	1.5	0.9

Table S2. Theoretical noise equivalent pressure (NEP) of detectors made of polyvinylidene difluoride (PVDF) and lead zirconate titanate (PZT)-based 1-3 composite piezoelectric materials.

2. Numerical modeling of relative imaging performance of PVDF and PZT arrays

To explore the impact of the spectral characteristics of PZT and PVDF detectors (Fig. S1) on optoacoustic tomography imaging quality, we conducted numerical K-wave modeling on a spatiotemporal grid that supported the maximum frequencies in the ultrasonic antennas' sensitivity spectrum up to 40 MHz. The digital phantom used was Shepp-Logan, representing the initial pressure distribution within the object roughly corresponding to the size of a rodent brain. The acoustic properties of the phantom object matched the ones for medium filling the space between the phantom and the antenna, with dispersion and attenuation effects not considered in the model. Signals from the digital phantom were recorded by an array of 512 independent arc-shaped elements with each element's aperture of 1.1 mm. The antenna's focal distance was 15 mm with an angular coverage of 180 degrees. Thus, we maintained the characteristic antenna geometry but limited the study to two-dimensional (cylindrical) modeling, as the available computer memory resources (40 Gb DDR6 VRAM + 256Gb DDR4 RAM) were insufficient for three-dimensional K-wave modeling [11] (Fig.S2).

To model different spectral sensitivity of PVDF and PZT arrays, the full frequency band (0-40 MHz) signals averaged by each of the 512 detector elements were noised (at a level of 25 or 40 dB relative to the peak signal level), and then passed through Fourier filters (Fig.S2) corresponding to the spectral sensitivity of PVDF or PZT elements. The tomographic reconstruction of the original image was performed using the time-reversal method implemented in the K-Wave software environment [11]. The quality of the reconstructed images was compared to the original based on metrics such as IMSE (image mean square error) and SSIM (structural similarity image metric).

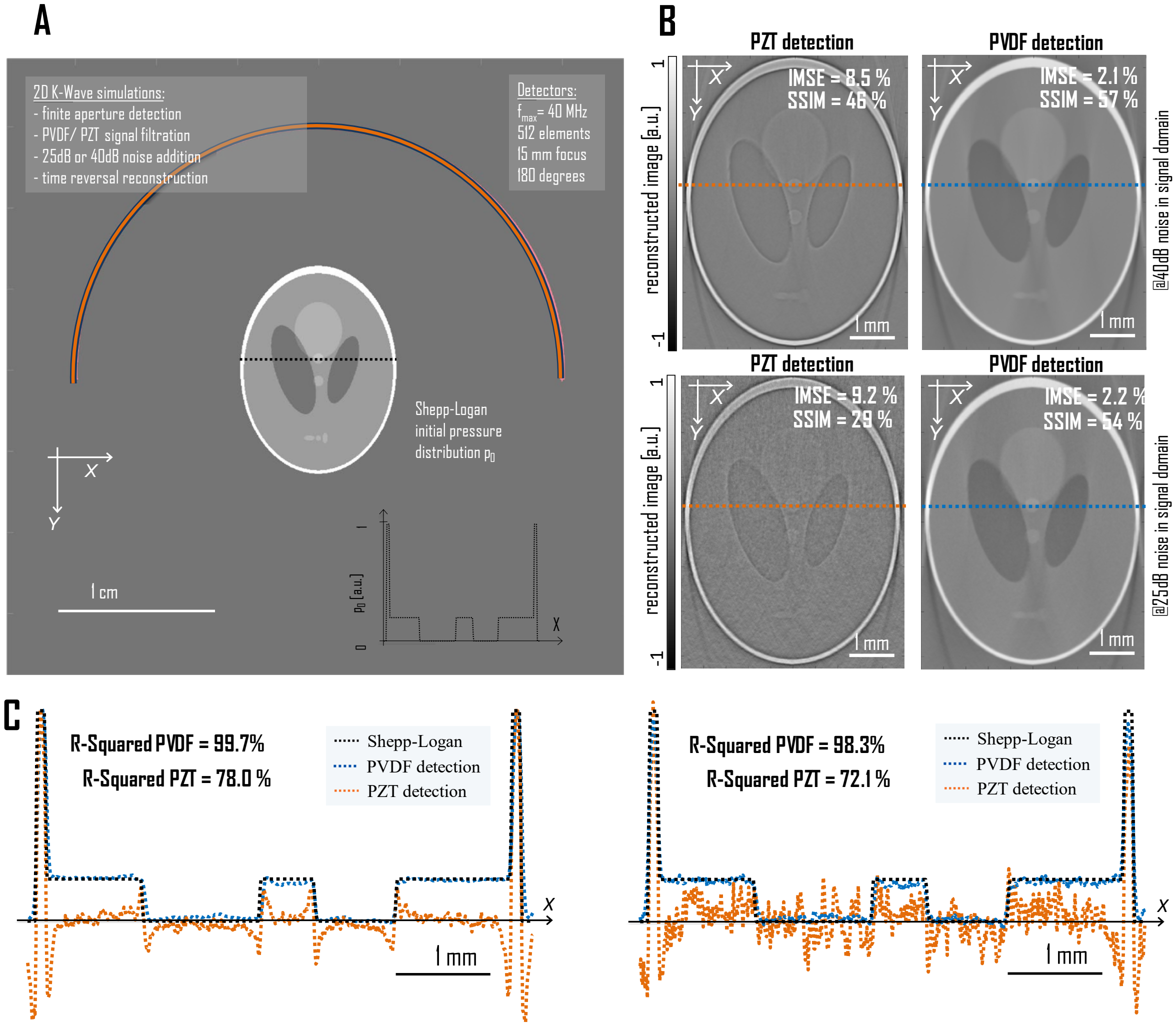

Figure S2. Detector array composed of PVDF elements outperforms PZT analog in precision for 2D optoacoustic tomography of a digital Shepp-Logan phantom. (A) Arrays characteristics, initial pressure distribution profile, and other parameters of K-Wave modeling. (B, C) Quantitative analysis of optoacoustic images reconstructed using PZT or PVDF array elements.

As can be seen from the corresponding images (Fig.S2B) and image profiles (Fig.S2C), the broadband sensitivity and high signal-to-noise ratio inherent to PVDF detectors result in a more accurate depiction of the Shepp-Logan phantom, especially within a noisy environment. Notably, the reconstructed images using PVDF detectors maintain high structural integrity and detail contrast, with the IMSE and R-Square metrics indicating less than 5% deviation from the original, even when subjected to noise levels of 25 to 40 dB in the signal domain.

3. Manufacture and characterization of a single-element PVDF prototype

The 512-element spherical antenna (Fig. 1A) was characterized through measurements carried out on a piston single-element prototype. As piezoelectric element of the single-element prototype, a 25 µm thick section of a biaxially stretched PVDF film (Precision Acoustics, UK) was used. A copper rod with 1.1 mm diameter was used as a signal electrode, and the ground electrode was deposited by a low-temperature method and consisted of a 50 nm chromium layer and a 100 nm gold layer.

In full accordance with the results of theoretical modeling (Fig. S1A), the small capacitance of the manufactured single-element prototype C ≈ 5 pF did not allow direct connection of the head to a standard transmission line with a characteristic impedance R = 50 Ω. This led to filtering of useful signals below the cutoff frequency $1/2\pi C_0 R > 0.5\text{GHz}$, and thus required an impedance matching amplifier

[10]. BF 998 double-gate field effect transistors (MOSFETs) with an operating bandwidth up to 1 GHz were used (Fig. 1A) to overcome bandwidth and sensitivity issues over a wide frequency band. Since only a relatively small voltage gain could be achieved with a single stage, a two-stage amplification circuit was used on the same transistor along with an emitter follower to match the amplifier with a 50 Ohm communication line [12]. An RC filter was installed between the stages of the amplifier, which suppressed the low-frequency part of the signal spectrum (below 300 kHz) to reduce low-frequency noise and amplifier overload due to the pyroelectric sensitivity of the PVDF film.

Experimentally, the sensitivity of the single-element prototype was determined by the relative method (Fig. S3). This involved comparing the spectra of OA signals from black polyethylene film for the prototype and the reference hydrophone (NH0500, Precision Acoustics, UK). When a thin light-absorbing film is irradiated with short laser pulses, short ultrasonic pulses are generated as a result of the OA effect. The duration of such ultrasonic pulses can reach several tens of nanoseconds, providing a wide frequency spectrum of the signal (Fig. S3A). By comparing the OA spectrum of the detector under study with the spectrum of the reference detector (Fig. S3B), sensitivity can readily be determined over a wide frequency range. The sensitivity at each frequency was determined as the ratio between the spectrum of the OA signals of the prototype and the spectrum obtained for the hydrophone NH0500, followed by multiplication by the known sensitivity of the hydrophone at the same frequency, namely,

$$Sensitivity_{prototype}(f)\left[\frac{\mu V}{Pa}\right] = \frac{Spectrum_{prototype}(f)[a.u.]}{Spectrum_{reference}(f)[a.u.]} \cdot Sensitivity_{reference}(f)\left[\frac{\mu V}{Pa}\right] \quad (20)$$

Both the piston single-element prototype and the commercial hydrophone were sequentially submerged in an immersion chamber containing distilled water, with a thin black film mounted parallel to them (Fig. S3C). Laser pulses (2 ns, 1 kHz) emitted from an ONDA532 diode-pumped laser (Bright Solutions, Italy), were directed onto the black film, providing a light spot diameter (≈10 mm) on the film surface that significantly exceeded the aperture of the detectors. Absorbing the laser pulses, the film generated optoacoustic waves crucial for sensitivity assessments. Signals from flat single-element PVDF prototype underwent amplification via an integrated amplifier (K=30), whereas reference hydrophone signals were enhanced through a two-tier amplification process (total amplification K=50). A 16-bit digitizer (CSE25216, GaGe, USA) with 500 MHz sampling digitized these signals.

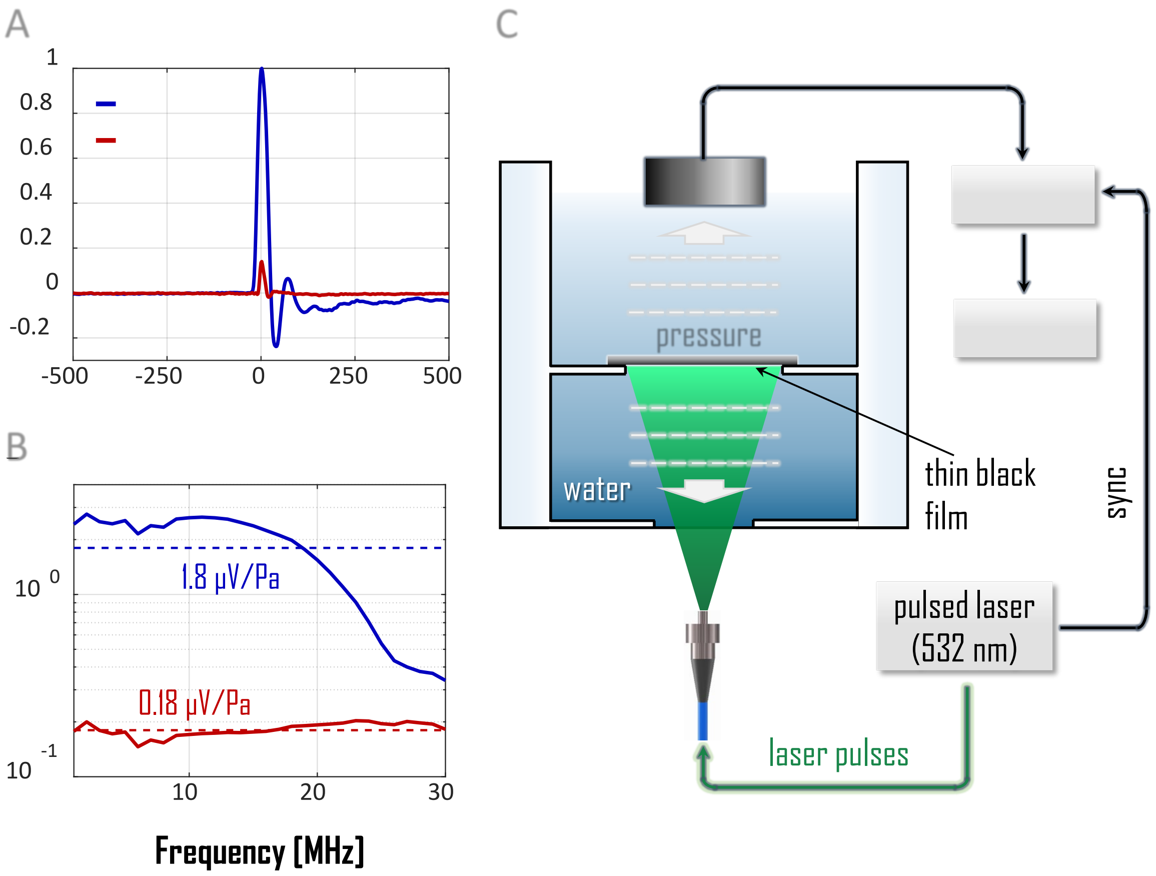

Figure S3. Experimental measurement of single-element PVDF prototype sensitivity. (A) Optoacoustic signals from a 15 µm thick black polyethylene film captured using single-element prototype and NH0500 hydrophone. (B) Comparison of the measured sensitivity of the single-element prototype to the hydrophone's reference sensitivity. (C) Experimental setup diagram.

Figure S3(A,B) shows the captured OA signals and sensitivities for the single-element prototype and the reference hydrophone. The reported average sensitivity of the piston prototype was in the range of 0.3–30 MHz was 1.8 µV/Pa. This was lower than the theoretically predicted value of 6.7 µV/Pa in a similar frequency range, a discrepancy attributed to intrinsic parasitic capacitance introduced during detector manufacturing.

	Single-element prototype		
**Cutoff frequency low f_1, MHz**	0.3	0.3	7
**Cutoff frequency high f_2, MHz**	7	30	
**Rms noise $_{[f_1,f_2]}$, µV**	4.9	9.6	8
**$\langle$Sensitivity$\rangle_{[f_1,f_2]}$, µV/Pa**	2.5	1.8	1.6
**Averaged NEP $\langle NEP \rangle_{[f_1,f_2]}$, Pa**	2	5.3	5

Table S3. Experimental noise equivalent pressure (NEP) of single-element PVDF prototype.

Subsequently, the thermal noise of the PVDF detector was examined across three frequency domains. Noise signals were digitized, filtered, and normalized by the amplification factor (K=30) to determine the NEP values, showcased in Table S3. With an average 0.3-30 MHz sensitivity of 1.8 µV/Pa and an effective RMS noise of 9.6 µV, the NEP for the spherical array components was determined as:

$$\langle NEP\rangle_{[0.3,30]\mathrm{MHz}} \approx \frac{9.6\ \mu V}{1.8\ \mu \frac{V}{Pa} \cdot \sqrt{30 \cdot 10^{6} Hz}} \approx 1 \cdot 10^{-3}\ Pa/\sqrt{Hz}. \quad (21)$$

SUPPLEMENTARY REFERENCES

1. Jaffe, H. & Berlincourt, D. A. Piezoelectric transducer materials. *Proceedings of the IEEE* **53** 1372–1386 (1965).

2. Mason, W. P. & Baerwald, H. Piezoelectric Crystals and Their Applications to Ultrasonics. *Physics Today* **4** 23–24 (1951).

3. Wild, M., Bring, M., Halvorsen, E., Hoff, L. & Hjelmervik, K. The challenge of distinguishing mechanical, electrical and piezoelectric losses. *The Journal of the Acoustical Society of America* **144** 2128–2134 (2018).

4. Mason, W.P. Electromechanical Transducers and Wave Filters. van Nostrand, New York (1948).

5. Domarkas, V.I., Kazhis, R.I.Y. Kontrolno-izmeritelnye pezoelektricheskie preobrazovateli [Verification and measurement piezoelectric transducers]. Mintis Publ, Vilnus (1974).

6. Olson, H.F. Dynamical analogies. van Nostrand, Princeton, 2729, (1958).

7. Uchino, K., Zhuang, Y., Ural, S.O. Loss determination methodology for a piezoelectric ceramic: new phenomenological theory and experimental proposals. *Journal of Advanced Dielectrics* **01** 17–31 (2011).

8. Nyquist, H. Thermal Agitation of Electric Charge in Conductors. *Physical Review* **32** 110–113 (1928).

9. Lundberg, K. *Noise sources in bulk CMOS.* Massachusetts Institute of Technology, Cambridge, unpublished PDF, p.10 (2002).

10. Subochev, P. et al. Wideband linear detector arrays for optoacoustic imaging based on polyvinylidene difluoride films. *Journal of Biomedical Optics* **23** 1 (2018).

11. Treeby, B. E. & Cox, B. T. k-Wave: MATLAB toolbox for the simulation and reconstruction of photoacoustic wave fields. *Journal of Biomedical Optics* **15** 021314 (2010).

12. Liu, Y.-H., Kurnikov, A., Li, W., Subochev, P. & Razansky, D. Highly sensitive miniature needle PVDF-TrFE ultrasound sensor for optoacoustic microscopy. *Advanced Photonics Nexus* **2** (2023).
